# Safety Concerns for the Management of End‐of‐Life Lithium‐Ion Batteries

**DOI:** 10.1002/gch2.202200049

**Published:** 2022-07-25

**Authors:** Zhuowen Chen, Abdullah Yildizbasi, Yan Wang, Joseph Sarkis

**Affiliations:** ^1^ School of Business Worcester Polytechnic Institute 100 Institute Road Worcester MA 01609 USA; ^2^ Department of Industrial Engineering Ankara Yıldırım Beyazıt University Ankara 06010 Turkey; ^3^ Department of Mechanical & Materials Engineering Worcester Polytechnic Institute 100 Institute Road Worcester MA 01609 USA; ^4^ LAMIH Laboratory Université Polytechnique Hauts‐de‐France Campus Mont Houy Valenciennes 59313 France

**Keywords:** circular economy, closed‐loop supply chain, end‐of‐life management, lithium‐ion batteries, safety

## Abstract

Lithium‐ion battery (LIB) usage is growing dramatically worldwide. Relatedly, there is a need for the management of end‐of‐life (EOL) LIBs. EOL requires closed‐loop systems and supply chains. Although many studies related to managing EOL in closed‐loop supply chains exist, one especially pernicious issue is overlooked—safety. This study seeks to address this major safety oversight for EOL LIBs using closed‐loop supply chains that are critical to a larger circular economy environment. The evaluation is completed along a technology–organization–environment (TOE) framework; potential research directions for mitigating safety issues are part of the analysis of this study. Specific and general research questions pertaining to secure management of EOL LIBs are put forward to help advance academic research. Practical concerns are also described for policymakers and organizations. This study reveals implications of these questions for the intersection of materials science, supply chain management, and fire‐protection engineering.

## Graphical abstract

Increasing end‐of‐life lithium‐ion battery quantities makes closed‐loop supply chains noteworthy to help maintain continuity of material sources. There are safety issues in these closed loop processes. Specific research questions are proposed based on the technology–organization–environment framework according to analysis of safety factors across various activities. Further evaluation of interactions and general research questions on risk mitigation are raised.

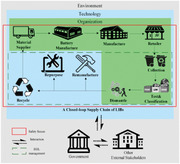

## 1 Introduction

The demand for lithium‐ion batteries (LIBs) is increasing greatly, as more products, especially electric vehicles, utilize them.^[^ ^1^ ^]^ Electric vehicles are expected to account for 11–28% of global vehicles in 2040.^[^ ^2^ ^]^ Increased LIB consumption will inevitably lead to growth in LIB waste and materials resource scarcities.^[^ ^3^ ^]^ The global LIB recycling market is expected to reach $11 billion by 2027.^[^ ^4^ ^]^ The amount of end‐of‐life (EOL) LIBs generated mainly from electric vehicles will reach 1336.5 GWh in 2040.^[^ ^5^ ^]^ These quantities and resource requirements necessitate minimizing LIB waste and mitigating resource scarcity through various circularity practices such as recycling, remanufacturing, and repurposing. These EOL practices to manage LIB resources and sustainability can be directly related to a broader circular economy philosophy.^[^ ^6^ ^]^

EOL management within a circular economic closed‐loop system is needed for a number of reasons. First, there will be 300 million electric cars on the roads by 2030, accounting for more than 60% of new cars, compared to 2020, when they accounted for only 4.6% of new cars.^[^ ^7^ ^]^ The EOL LIBs, mainly those from electric vehicles, still have high utilization value. Electric‐vehicle LIBs are expected to last ≈8–10 years and should be replaced when the capacity loss reaches 20%.^[^ ^8^ ^]^ From an economic viewpoint, remanufacturing LIBs represents a 40% cost savings when compared to producing batteries from virgin materials.^[^ ^9^ ^]^ LIBs retired from electric vehicles are also widely believed to be suitable for lower requirement applications, a repurpose aspect, such as for energy storage.^[^ ^10^ ^]^

Second, the supply of raw materials for LIBs can be erratic.^[^ ^11^ ^]^ The main LIB materials include lithium, manganese, graphite, cobalt, and nickel. These commodity‐material markets are dominated by a small number of countries: 67% of graphite production is from China; 59% of cobalt production is from the Congo; 44% of lithium production is from Australia.^[^ ^12^ ^]^ LIB supply chain resilience can be improved through EOL LIBs and circular economy practices.

Third, EOL LIB management can reduce emissions and resource usage. LIBs without EOL management are harmful to the environment and are a significant motivation for this practice.^[^ ^13^ ^]^ Cobalt and lithium, the key materials for LIBs, are scarce, and most EOL LIBs are improperly disposed of rather than collected, which causes water and soil pollution.^[^ ^14^ ^]^

**Figure 1:**
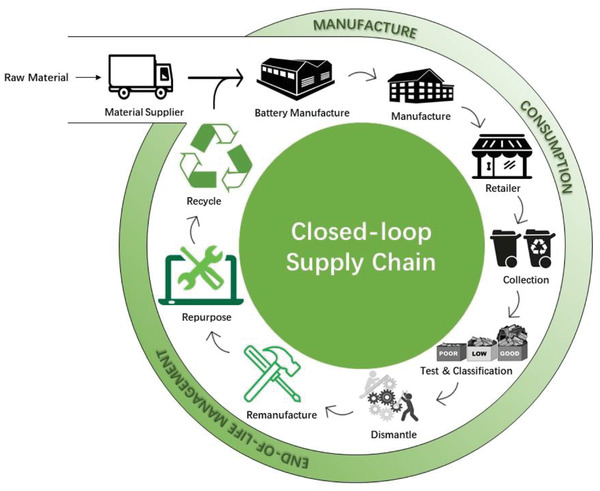
A closed‐loop supply chain for lithium‐ion batteries.

Overall, it makes business and environmental sense to consider LIB closed‐loop supply chains (CLSC) within the context of a circular economy (**Figure** **1**). Recycling, for example, can reduce manufacturing costs by 25.6–36.6%, water consumption by 30.1–41.2%, and greenhouse gas emissions by 29.3–38.2%.^[^ ^15^ ^]^ One under‐investigated, but critical dimension in the EOL LIB supply chain management, is safety, which is an important social sustainability dimension. It is a primary challenge for large‐scale application in the LIB industry,^[^ ^8^ ^]^ with surprisingly minimal investigation. LIB safety risks can arise anywhere in the supply chain, and EOL management in the CLSC is not immune from these safety issues.^[^ ^16^ ^]^

A litany of recent safety incidents further supports the need for investigation of LIB supply chain safety. The UK's Environmental Services Association found that of 670 fires recorded between April 2019 and March 2020, 145 were directly attributable to LIBs and another 112 were suspected.^[^ ^17^ ^]^ There have been many cases of garbage trucks catching fire due to improper LIB disposal. The UK's Environmental Services Association reported that the UK waste industry faced 201 fires caused by LIBs in 2021.^[^ ^18^ ^]^ The California Product Stewardship Council found that 40% of waste management facility fires were caused by LIBs.^[^ ^19^ ^]^ LIB‐related safety accidents have caused substantive economic losses and casualties, as well as a negative impact on the environment. LIBs account for almost half (48%) of all waste fires in the UK each year, costing that economy an estimated £158 million a year.^[^ ^18^ ^]^ LIB fires also cost the U.S. and Canada more than $1.2 billion annually.^[^ ^17^ ^]^ Safety issues negatively influence economic, environmental, and social aspects of the whole supply chain, and are likely to increase as LIB usage increases. Investigating safety issues within the LIB CLSC can broadly benefit safety research, in general EOL management and circular economy practices, with both sets of practices growing in importance for sustainability research but not effectively studied from safety perspectives.

This study focuses on the reverse flow within the CLSC, as this reverse flow is most closely aligned with EOL LIBs. The objective is to introduce and evaluate safety factors that affect LIB safety at each stage of the reverse‐flow portion of the supply chain. The work utilizes the technology–organization–environment (TOE) framework, to help categorize safety factors and provide a foundation for future research in this area, the second objective of this study.

Given these objectives, below is a summary of this study's contributions:

1. Providing further insight into LIB supply chains from the perspectives of circular economy and EOL management.
2. Identifying and raising awareness in scholarly research on LIB CLSC safety concerns specifically, and safety issues in supply chain and circular economy generally.
3. Proposing potential research directions and a framework for investigations into safety problems with EOL LIB in the closed‐loop supply chain.

The remainder of this paper begins with Section 2, which reviews LIB CLSC and EOL management. Potential risks in the supply chain are also identified in this section. Safety dimensions across different CLSC processes are identified and integrated in a TOE framework in Section 3. Section 4 introduces a research agenda with specific and general research questions about EOL LIB safety issues, using the TOE framework. Practical and research implications of the findings appear in Section 5. Section 6 is a conclusion that summarizes the study, limitations, and future steps.

## 2 Background

### 2.1 Closed‐Loop Supply Chain and End‐Of‐Life Management

Increased LIB demand and sourcing issues have resulted in significant attention on supply chain activities.^[^ ^20^ ^]^ A supply chain refers to an integrated process involving suppliers, manufacturers, distributors, retailers, and reverse logistics, with the closing of the supply chain loop, depending on recycling, repurposing, and remanufacturing (Figure 1).^[^ ^21^ ^]^ Major supply chain uncertainties include raw material supply and EOL management.

CLSC combines forward and reverse activity flows to improve economic and environmental benefits.^[^ ^22^ ^]^ It combines a forward (material supplier, manufacturer, retailer, and user) and a reverse logistics and supply chain (collection, testing, classification, dismantling, remanufacturing, repurposing, and recycling), as shown in Figure 1. The forward supply chain refers to the combination of processes that meet end‐user customer requirements.^[^ ^23^ ^]^ The reverse supply chain generally starts with the end‐users, collecting the used products, then managing EOL products through different methods such as repurposing, remanufacturing, and recycling.^[^ ^23^ ^]^ CLSC has been widely recognized in academic research and practical application in different fields and is central to the broader circular economy philosophy.^[^ ^24^ ^]^ In this study, EOL LIB management will focus on the reverse portion of the CLSC. The importance of LIB CLSC research is evidenced by the economic value of EOL LIBs and risks in managing the various processing environments of EOL management.

About 40% of the economic value of EOL LIBs arises from cathode materials.^[^ ^12^ ^]^ The rapid rise in lithium and cobalt prices has made EOL LIB management more profitable. Additionally, reverse logistics practices including remanufacturing, repurposing, and recycling play an important role^[^ ^25^ ^]^ both to reduce the use of natural resources and increase economic efficiency.^[^ ^26^ ^]^ In addition, EOL LIB safety concerns exist, with numerous fires and explosions resulting in casualties and economic losses. LIB EOL practices can inform broader circular economy concerns, where safety issues have rarely been emphasized in circular economy practices.^[^ ^27^ ^]^

### 2.2 Safety Concerns Across Processing Steps Within End‐Of‐Life Management

EOL LIB requires management of reverse product and materials flows. Collection is the first step in EOL management in the reverse logistics segment of the CLSC, but the process is limited in various countries. For example, the EOL LIB collection and recycling rate in China is less than 10%.^[^ ^28^ ^]^ Improper disposal as an alternative EOL practice to circularity practices such as in landfills and incineration, causes soil, air, and groundwater pollution, which can also result in fire and other safety accidents.^[^ ^29^ ^]^ Given limited LIB raw material resources, many countries are formulating relevant collection and take‐back regulations over the LIB lifecycle to lessen disposal of this valuable EOL resource.^[^ ^30^ ^]^ These pressures will result in further concern and the necessity to manage the reverse supply chain, where safety concerns still exist.

Collected EOL LIBs, in the next phase of the reverse supply chain, may be tested and classified by their state, safety, and remaining utilization. Testing and classification of collected EOL LIBs includes rapidly discharging the LIBs and requires meeting safety standards.^[^ ^31^ ^]^ Improper testing operation and unsafe operating conditions can easily lead to fires and explosions.^[^ ^31^ ^]^ Unstandardized classification can also damage the batteries and increase the risk of battery aging and thermal runaway.^[^ ^8^ ^]^

Dismantling is another possible activity for EOL LIB management. This step may include disassembly, crushing, and separation after collection. Typical products with LIBs that require dismantling include laptops, mobile phones, and EVs. These products are dismantled manually.^[^ ^32^ ^]^ Therefore, unstandardized operations, shortage of safety precautions, or unstandardized emergency responses are potential safety hazards, due to the requirement for significant human involvement.

EOL LIBs, in a CLSC, can be remanufactured, repurposed, or recycled. LIBs with enough capacity and remaining value can be remanufactured. In remanufacturing, some damaged cells can be replaced by new cells or qualified cells that come from other EOL LIB packs after diagnosis.^[^ ^33^ ^]^ The difference between remanufacturing and repurposing is that repurposing might need to apply new software and hardware for different applications. Repurposed LIBs are generally used for energy storage, for example, stationary applications, which need lower current density than the battery packs used in EVs.^[^ ^34^ ^]^ Recycling is the broadest and most frequent circular economic practice and CLSC practice for EOL LIBs.^[^ ^12^ ^]^ Two current industry recycling practices are pyrometallurgical and hydrometallurgical processes, and another method under lab development is direct recycling processes.^[^ ^12^ ^]^ The variety of activities across stages and practices of EOL LIB management increases safety risks. These safety issues are critical obstacles to EOL LIB CLSC processes, but they are also generally a concern for many other materials as well.

Safety issues in the LIB CLSC exist at all stages including movement, transportation, storage, inventory, and operation.^[^ ^35^ ^]^ In these activities, a number of issues may arise. For example, ambient temperature and humidity can affect LIB stability. Sealing and isolation conditions during LIB storage need to be considered. Another example of a safety issue is pressure change during transportation, which might occur in air cargo. Train or highway transportation includes risks of collision, impact, or impaling. The testing and dismantling process stage requires substantial manual operations and human involvement, which increases the risk of improper operation.

Previous studies considered LIB safety incidents. LIBs contain active materials such as lithium and flammable solvents, which can easily lead to thermal runaway beyond the safe operating range.^[^ ^16^, ^36^ ^]^ Even under normal operating conditions, it is possible for adverse reactions to take place, due to elevated temperatures.^[^ ^37^ ^]^ LIBs have been blamed for fires and explosions on planes and in airports in various countries.^[^ ^38^ ^]^ There are many cases of aviation accidents caused by hidden cargo containing LIBs.^[^ ^39^ ^]^ EOL batteries have also been the cause of fire accidents at the waste‐treatment stage, which poses a threat to the entire waste‐management sector.^[^ ^19^ ^]^

These examples of safety issues are only a partial list of extensive known and potential safety risks. In EOL management, LIBs require multiple processing steps, with varying safety issues going well beyond these examples. This evidence and potential future occurrences with new evidence are reasons for delving into and considering safety in LIB EOL situations.

Most literature focuses on the safety design of LIBs,^[^ ^40^ ^]^ and current literature also investigates LIB safety tests, because of explosions and fire accidents.^[^ ^37^, ^41^ ^]^ However, few articles pay attention to the safety problems from the perspective of CLSC and EOL management. This research study highlights the safety problems, mainly fire and explosive accidents, of the EOL LIBs in the reverse supply chain. It differs from previous studies in the inclusion of discussion about potential research directions, using the TOE framework as a theoretical framework for the investigation of safety issues. The results and framework for EOL LIBs provide initial insights for broader safety research in reverse logistics, CLSC, and circular economy practices.

## 3 Safety Issues: A Technology–Organization–Environment Perspective

### 3.1 Safety Issues in the End‐of‐Life Lithium‐Ion Battery Supply Chain

This section categorizes and evaluates incident sources of safety issues for LIBs. **Table** **1** presents the initial categorization along the main safety dimensions. LIB safety issues can be classified into mechanical, electrical, chemical, and thermal abuse categories.^[^ ^42^ ^]^ This section also expands on these issues by introducing a social dimension and identifying various sub‐dimensions.

**Table 1:** Categorization of safety issues

Main Dimension	Sub‐dimension	Short descriptions	Supporting literature
Electrical	Overcharge and overdischarge	Batteries with different charging and discharging times vary in capacity retention, aging, inner structure, and safety condition. Only fully discharged batteries with no physical damage are considered safe devices.	[37, 43, 44, 45, 46, 47, 48, 49, 50, 51]
–	Packaging concerns	Isolation conditions for inner and outer packages will affect the stability of batteries, such as the existence of inner tape for the package and the sealing conditions of the external package.	[52, 53]
Mechanical	Ambient pressures	The thermal safety of LIBs is significantly affected by different ambient pressures caused by different transportation and storage conditions. For instance, the heat release rate is changed by the altitude.	[43, 54, 55]
–	Physical damages	Improper storage and transportation might cause damages to LIBs such as impact, squeeze, vibration, penetration that led to electrolyte leaking, short circuit, structure crack, etc.	[56]
Chemical	Battery materials usages	Batteries using different active materials, electrolytes, and separators can have various end‐of‐life stability performance. The Li‐ion intercalation and deintercalation processes in battery life led to different volume changes with different materials, resulting in variable safety issues which may cause different hazards to workers and the environment.	[57, 58, 59, 60, 61]
	Materials usages for processes	Workers may be exposed to hazardous substances generated by different functional chemical materials used to dispose EOL batteries, which may cause occupational injuries.	[51, 61, 62, 63]
Thermal	Ambient temperatures	The temperature change will trigger unexpected reactions. Temperatures over 40 °C can cause thermal runaway, short circuits, volume expansion, and speedup of side reactions, leading to combustion or explosion.	[64, 65, 66, 67, 68, 69, 70]
–	Humidity	High humidity levels will change the outside condition of batteries. Micro‐short circuiting, irreversible self‐discharging, and structure change of materials are more likely to take place as the humidity grows.	[46, 71, 72, 73, 74]
Social	Untrained staffs	The participation of untrained staff is common in some recycling companies, which leads to increased risk throughout the whole supply chain.	[1]
–	Delayed emergency responses	Fires caused by LIBs require specialized methods to extinguish them. Current emergency measures are not up to scratch and need to be built up.	[75]
–	Flawed regulations	Regulations about LIB safety are still incomplete in various countries, which leads to many irregulated operation processes and causes a lot of incidents. Meanwhile, it is important to harmonize labeling requirements and terminology.	[16, 76, 77]
–	Oblivious users	The cases of fire caused by improper disposal and unreasonable use of LIBs happen a lot.	[16, 78, 79]
–	Hazardous pollutants	The recycling process will produce harmful substances and cause environmental pollution. Burning LIB waste might lead to the release of toxic gas. Hydrometallurgy requires acid to dissolve the components, producing harmful wastewater. The toxic metal in batteries can cause injury to workers, children, and pregnant women.	[80, 81, 82, 83, 84, 85, 86, 87]

Electrical safety issues include overcharge or overdischarge, and packaging concerns. Usually, these elements are very much related to the design of the batteries and their packaging. The mechanical dimension mainly refers to physical damages to batteries. The usage of battery materials and the chemicals required for EOL management processing are the two main sources of chemical safety hazards. The thermal dimension includes external temperature and humidity concerns during collection, transportation, and storage. Each of the examples presented in Table 1 relates to supply chain and EOL operations that exist in the LIB CLSC.

There are five safety issues within the social dimension: 1) Untrained staff working at different stages of the EOL LIB management, especially in dismantling and collecting phases, which may result in occupational injuries due to handlers’ lack of process expertise. 2) Stakeholders including enterprises, communities, and the government, who may not provide an adequate emergency response to fire and explosion accidents caused by LIBs. As an example, recycling facilities generally lack safety design. 3) LIB regulations and standards are flawed, especially EOL LIBs with gaps in specific regulatory policies. 4) The lack of safety awareness among users about LIBs; improper disposal of EOL LIBs is the primary reason for pollution and hazardous incidents. 5) Related pollutants directly from EOL LIBs and indirectly through accidents. Each of these safety concerns will affect the economic, environmental, and social dimensions of EOL LIB management.

The TOE framework for EOL LIB safety management, discussed in the next section, sets the stage for a broader set of research questions pertaining to this area.

### 3.2 The Technology–Organization–Environment Framework for Safety Issues

#### 3.2.1 The Technology–Organization–Environment Links to Closed–Loop Supply Chain Activities

**Figure 2:**
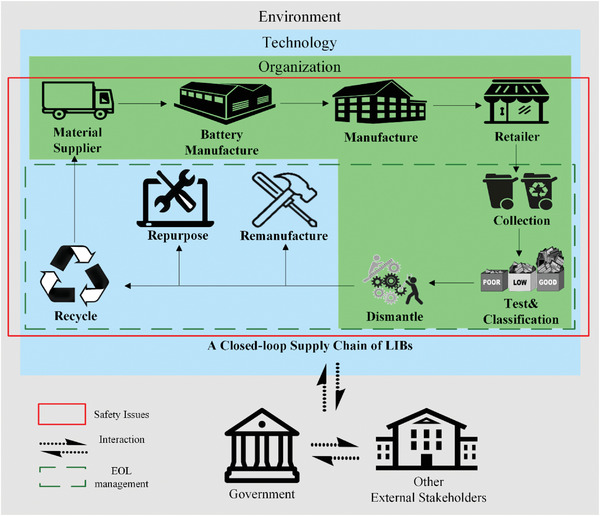
The closed‐loop supply chain of Lithium‐ion batteries based on the technology‐organization‐environment framework.

The TOE framework is used to evaluate safety issues from technological, organizational, and environmental dimensions.^[^ ^88^ ^]^ **Figure** **2** provides an overview of these relationships across the supply chain by restructuring Figure 1 along the TOE dimensions.

Many perspectives can be provided for the supply chain, given that this analysis focuses on CLSC and EOL; however, this research concentrates on a high‐level perspective of the forward supply chain in Figure 2, while restructuring Figure 1. Supply chain materials suppliers, whether supplies are sourced through the CLSC or from virgin materials or extractors, appear in the upper‐left portion of the closed‐loop supply chain in Figure 2. Forward LIB supply chains include battery‐specific manufacturers as suppliers to original equipment manufacturers (OEMs) such as vehicles or electronics manufacturers. These products are then sold to consumers (industrial consumers may be included) and retailers. Products and materials are then consumed. As they reach their EOL, they return into the industrial supply chain through reverse supply chain and logistics activities.

In Figure 2, a dashed line sub‐boundary illustrates the EOL process boundaries. Collection, testing and classification, and dismantling processes have organizational safety concerns, due to their manual operations, although technological concerns do exist there and in previous stages. Technological safety concerns tend to dominate when it comes to final disposition, based on the circular economy practices of the Re's—repurpose, remanufacture, and recycle. Technological safety concerns occur throughout the entire CLSC, beyond the supply chain organizational, and heavily dependent manual processes.

Environmental dimensions include actions and activities by government and other external stakeholders, which also include interactions within the internal stakeholders. The federal government plays an interactive role with management and the relationships to internal stakeholders and other external stakeholders. For example, the laws and regulations set by governmental authorities could be valid for each of the other stakeholders. Figure 2 makes clear that safety issues occur throughout the entire supply chain, with the solid red line box; and in this context, the environmental safety issues and relationships play a role throughout the supply chain. The TOE elements that have more specific relationships to the dimensions identified in previous sections are described later in this section.

By understanding the connections between safety issues in the supply chain and the TOE framework through Figure 2, it is possible to classify each safety factor along these perspectives. There may be overlaps, as some issues cover multiple dimensions, but the research has established an initial home for each of the dimensions. These dimensions and TOE categories are described in more detail below.

#### 3.2.2 The Technology–Organization–Environment Framework Links to Lithium‐Ion Battery Safety Concerns

Table 1 introduces and summarizes 11 sub‐dimensional safety factors for CLSC. These safety factors can be classified into three categories, according to the TOE framework in **Table** **2**, with external stakeholders added as the twelfth environment category sub‐dimension. This additional dimension is due to the multiple stakeholders involved along the supply chain who are external to the organization.

**Table 2:** Category of safety issues based on technology‐organization‐environment framework

Main dimension	Sub‐dimension	TOE framework
Electrical	Overcharge and overdischarge	Technology
–	Packaging concerns	–
Mechanical	Ambient pressure	–
–	Physical damages	–
Chemical	Battery materials usage	–
–	Materials usages for processes	–
Thermal	Ambient temperatures	–
–	Humidity	–
Social	Untrained staff	Organization
–	Delayed emergency responses	–
–	Oblivious users	–
–	Flawed regulations	Environment
–	Hazardous pollutants	–
–	External stakeholders	–

Technological safety concerns may utilize existing technology from within and outside the organization.^[^ ^89^ ^]^ EOL LIB safety concerns and improvements of CLSC are based on new technologies and technical designs and innovations and typically may be used to contribute or address electrical, mechanical, chemical, and thermal issues.

The organizational dimension of the TOE framework refers to internal organizational concerns, including corporate culture, business processes, business practices, and management structures.^[^ ^90^ ^]^ This dimension may include problems related to internal management, human resources, and staff quality and routines.^[^ ^91^ ^]^ For example, poor staff operations and management, due to poor training, can lead to fire accidents in the very manual CLSC processes of collection and disassembly. Managerial and organizational resources may also play a role. As an example, small battery recycling companies with limited or informal safety management processes and poor training typically have more violations and fewer safety protection measures.^[^ ^92^ ^]^ Understanding these organizational issues can help reduce the probability of fire and explosion accidents, especially through formal standardized operating procedures and training. Organizational policies and management should reflect awareness of important EOL LIB CLSC safety issues.

The environmental dimension focuses on issues outside the organization, but not necessarily technologically oriented concerns. The larger societal community, culture, policy, competitors, and public infrastructure are examples of environmental factors that may influence LIB EOL safety concerns.^[^ ^90^ ^]^ Typical environmental EOL LIB safety problems would include imperfect laws and regulations, incomplete monitoring and auditing by external agencies, and a relatively immature supply chain, where various players in the supply chain outside the organization are not knowledgeable in managing safety concerns. Some of these dimensions are included in the present analysis, which provides some of the major difficulties, especially those related to stakeholders and government interactions. Legal actions, liability, insurance companies, and consumer‐advocacy groups, are additional and specific examples that can extend the external environmental dimensions even further.

The next section uses the TOE framework to develop a research agenda for investigating EOL LIB safety issues across multiple perspectives. Research in this area is almost non‐existent and relates importantly to CLSC, by extension, the circular economy, safety concerns. The history of CLSC and the circular economy has been focused primarily on environmental sustainability issues. Expanding the focus to social concerns, especially safety, contributes greatly to understanding the relatively nascent social sustainability concerns in CLSC^[^ ^93^ ^]^

## 4 Research Questions for End‐of‐Life Lithium‐Ion Battery Safety Concerns

The TOE framework interpretation of EOL LIB safety issues allows us to further consider the internal and shared interactions of the three TOE perspectives. This section synthesizes and extends observations from the previous sections of this paper, resulting in an initial series of specific research questions (SRQs) associated with a particular safety issue as well as general research questions (GRQs) associated with cross‐cutting issues. These research questions are based on the TOE framework and set a foundation for a research agenda for this inchoate field.

### 4.1 Specific Research Questions

Research addressing and investigating specific safety concerns is diverse and influenced by the technical, practical, or operational differences of safety issue sources. For example, the main causes (sources) of safety issues included within electrical, mechanical, and thermal dimensions are related to the characteristics of LIB materials. Thus, technological aspects can be used to ameliorate battery‐ and related product‐safety design issues. There are significant materials and scientific research requirements in order to make LIBs during EOL activities more stable and less susceptible to outside influences and shocks. Social, managerial, resource‐based, and behavioral issues also arise and set the stage for multi‐disciplinary research. Internal organizational and external environmental investigations can be helpful for addressing safety problems related to social and behavioral concerns; for example, research into management, operations, and legal matters can help uncover important safety management issues for EOL LIBs.

**Table** **3** summarizes exemplary SRQs, shown in the third column, aligned with each safety sub‐dimension. For each of these safety sub‐dimensions, possible research questions that extend existing research are presented. Support for why these exemplary research questions are valid and feasible opportunities are briefly described in the final column of Table 3.

**Table 3:** Specific research questions for safety issues based on the technology–organization–environment framework

TOE dimension	Safety issues	Exemplary specific research questions	Brief description
Technology	Overcharge and overdischarge	Can fuse‐likely material be applied into the battery system to overcome the overcharge and overdischarge problem by modifying the inner circuit? Can we detect the volume change for the whole cell or phase transformation of the material to mitigate the influence?	When overcharge and overdischarge take place, the fuse‐likely material might be heated and melt, as it cuts off the circuit to prevent further issues. Overcharge will also cause O_2_ evaluation and irreversible reactions for the cathode materials.^[^ ^94^ ^]^
–	Packaging concerns	Can new battery containers or casing material be useful to prevent risks?	The changed design of the battery casing^[^ ^95^ ^]^ might be effective to ensure the positive and negative electrodes are separated when the batteries are placed without isolation.
–	Ambient pressure	Can battery casing or packaging be redesigned to make LIBs less vulnerable to external physical damages and pressure? What does a safety‐based design mean for circular economy (CLSC) practices?	The effect from extra‐physical impacts differs in different materials.^[^ ^96^ ^]^ The risk of safety issues will increase substantially, while the battery containers face physical damages.
–	Physical damages		
–	Battery materials usage	Is it necessary to examine the impact of chemical material composition on the safety of end‐of‐life batteries when designing LIBs? Is it possible to mitigate safety risks from a chemical materials standpoint while still reaping economic and environmental benefits?	Chemical materials have a considerable impact on the safety of EOL LIBs. Therefore, considering the usage of materials in battery design from the view of EOL management safety is meaningful.
–	Materials usages for processes	Does the use of safer materials in the EOL management process affect recycling efficiency? How do the advantages of better EOL management processes stack up against the advantages of EOL management technologies?	Chemical components used in LIB end‐of‐life management could constitute safety risks. It's worthwhile to use technical and managerial tools to address these safety concerns.
–	Ambient temperatures	Is it possible to use technology to monitor the temperature and humidity condition of the battery, so that hazards can be avoided or predisposed? Can sensor technology contribute to safety monitoring and sensing? Can these monitoring devices serve multiple purposes benefits to circular economy practices?	Maintaining a moderate working‐state and timely transformation is important.^[^ ^97^ ^]^ Information management and sensor technology armed nanotechnology might be critical for transparency, traceability and visibility to manage safety along the supply chain.
–	Humidity		
Organization	Untrained staff	What are organizational tradeoffs for staff training? How much economic benefit and environmental improvement can human‐resource‐safety awareness bring to the supply chain?	Routine safety training for employees, such as updating state of art in time, might be effective in reducing safety problems in the practical operating process, as battery technology changes constantly.^[^ ^1^ ^]^
–	Delayed emergency responses	Do companies have standardized operations and emergency response requirements for fire and explosion incidents?	LIBs in different products have different states, especially EOL LIBs.^[^ ^12^ ^]^ Thorough emergency treatment for LIB‐related accidents may make sense to reduce the cost of accidents.
–	Oblivious users	To what extent can user awareness reduce particular safety issues in the supply chain? Are oblivious users a barrier to collecting EOL LIBs?	The safety awareness includes the safety range of using LIBs and the correct disposal of EOL products. For instance, fires and explosions caused by dumping EOL LIBs in the general garbage could be reduced by improving users’ safety awareness.^[^ ^98^ ^]^
Environment	Flawed regulations	Can standardized hazard labeling reduce risks during processing? To what extent can government supervision and improvement of regulations on the LIB supply chain reduce safety issues? What are the active impacts on the financial performance of the organization after establishing sound regulations?	The EOL LIB management varies greatly in different countries and regions.^[^ ^98^, ^99^ ^]^ The non‐standard recovery and treatment of LIBs by some small recycling enterprises might lead to safety accidents.^[^ ^98^ ^]^ Whether the unified labeling of identification and the standardization of processing specifications succeed in reducing risks is worth studying.^[^ ^75^ ^]^
–	Hazardous pollutants	How can the environmental hazards be reduced by managerial and technical improvement in battery design? Will new supply chains and technologies bring additional environmental, economic, and social problems?	The development of advanced technology provides opportunities and challenges to the environment and society.^[^ ^100^ ^]^ Alternative environmentally friendly materials might be invented as a new generation of LIBs. EOL management is intended to be effective in reducing pollution from batteries.^[^ ^8^ ^]^
–	External stakeholders	How can different stakeholders such as competitors, supply chain partners, and standards organizations, such as Underwriters Laboratory, influence the safety issues associated with EOL LIBs?	As LIB CLSC becomes more and more complex, the increased number of stakeholders, such as the emergence of small and medium‐sized battery collection companies, can influence supply chain safety issues. Competitor battery designs introduce change such as the development of hydrogen‐ion cells.^[^ ^101^ ^]^ How these situations increase or decrease the risks of the LIBs supply chain is worth discussing.

The technological segment of the table includes such examples as research, development, and application of sensor technology, alternative stable materials, battery casing and packaging design technology, and monitoring and tracking technology as areas for fruitful technology‐based research. From an organizational perspective, research may cover business, managerial, and economic concerns and research to help improve safety for EOL LIBs in the CLSC. For example, asking about the most effective ways and implications of CLSC safety: investing in training employees, optimizing emergency response systems, and/or creating safety awareness among participants in the closed‐loop supply chain. Relating organizational activities to safety outcomes is an important aspect of determining what is and is not effective.

In addition, along the external environment dimension, it is crucial to consider the role of governments and organizations in improving standards, laws, and regulations, and in reducing pollution. These stakeholders and institutional pressures may serve as motivation for actors within the EOL LIB supply chain to address safety concerns. Determining which standards and rules for institutions and which safety sources are effective for addressing safety concerns is part of these research requirements.

Furthermore, SRQs have interdependent implications related to each other; as an example, the research on technology can also affect the improvements of research in the organizational and environmental areas. These cross‐cutting issues are addressed by introducing a series of general research questions and directions in the next section.

### 4.2 General Research Questions

The GRQs arise from general patterns and interrelationships from the SRQs, and the technological, organizational, and environmental dimensions within CLSC activities of EOL LIB. Below are two sets of GRQs for each of the technical, organizational, and environmental dimensions. These include technical development and management, organizational design and management, and current and future external environmental groupings. Overall, there are six major sets of GRQs, with exemplary research questions in each set. This section delineates some of these research areas with, where appropriate, representative citations from previous studies.

#### Technical Development (GRQ1)

LIB technology is evolving and progressing. In LIB design,^[^ ^56^, ^75^ ^]^ can the development and use of new technological innovations, for example, solid‐state LIBs^[^ ^102^ ^]^ mitigate EOL safety problems caused by existing liquid‐based LIBs? Additionally, from the perspective of other emergent process and information technologies, can EOL LIB safety concerns be mitigated by applying multi‐stakeholder, information‐based industry 4.0 technology^[^ ^103^ ^]^ including sensors, big data analysis, and blockchain technology? Can these technologies help build scale operations while monitoring safety?

Technical development and innovation can occur anywhere in the LIB CLSC, and questions about how to manage these technologies will arise. GRQ2 focuses on technical management from the viewpoint of the economy, the environment, and society.

#### Technical Management (GRQ2)

What are the interactions and relationships that occur when utilizing these technologies, considering the EOL LIB tensions, synergies, and tradeoffs related to economic, environmental, and social benefits, along with safety concerns? Will there be a reduction in battery recycling rates due to increased safety and liability costs for emergent LIB technologies? Are there tradeoffs between these benefits or co‐benefits that may support both technological safety and circular economy (CLSC and EOL) concerns? What new safety precautions are needed as new technologies emerge? What safety sub‐dimensions are most affected? What new risks will arise from the use of new technologies?

With the adoption of new techniques in different process steps, part of the supply chain may be changed.^[^ ^104^ ^]^ Therefore, the relationship between the organization and safety issues in GRQ3 is considered next.

#### Organization Design (GRQ3)

Can EOL LIB safety issues be mitigated by redesigned LIB CLSC activities?^[^ ^105^ ^]^ What are the barriers and requirements for different organizations, when redesigning the supply chain, especially the CLSC? What is the impact of safety issues on the redesigned supply chain? Can supply chain safety risks associated with EOL LIBs be reduced by merging or splitting process steps? For example, collecting, testing, sorting, dismantling, remanufacturing, repurposing, and recycling steps might be merged in the closed‐loop supply chain and be more effective for EOL LIB management. Large battery manufacturers generally have the ability and resources to educate employees about safety and to monitor their products more easily, but how can smaller organizations do this?

Redesigning organizational design dimensions of the supply chain can be supported by organizational management of the CLSC and EOL LIBs. The frequency and impact of safety occurrences vary across CLSC operational activities, and managerial practices and policies can mitigate safety risks.

#### Organization Management (GRQ4)

How do safety issues affect different CLSC stages for EOL LIBs? How do safety concerns for untrained employees on transportation, storage, and dismantling processes differ? Which CLSC process step has the greatest relationship to safety concerns? Which one has the least relationship? Does measuring—performance measures and metrics development—the influence of safety issues at different CLSC stages in the LIB CLSC help stakeholders optimize safety repercussions? How can internal and external organizational stakeholders aid in mitigating safety issues for EOL LIBs?

The external‐to‐the‐organization environmental perspective includes current and future environmental research requirements; that is, there are current, immediate concerns, and potential future and emergent concerns that can occur externally. The massive use of LIBs makes both the internal supply chain and the external environment more diverse and complex.^[^ ^106^ ^]^ The following first general research question for external environmental factors focuses on CLSC EOL LIB safety relationships to external stakeholders such as governments, material suppliers, and competitors.

#### Current Environment (GRQ5)

How do external‐to‐the‐organization environmental factors, including policy, society, competitors (such as the development of cyanide‐ion batteries and other batteries), and other stakeholders^[^ ^107^ ^]^ (such as government regulation and raw material suppliers) affect supply chain and EOL LIB safety? Policy‐related safety factors include incomplete EOL collection policies and inconsistent safety standards and labels in different regions, resulting in incorrect identification for the status of LIB. The origin of LIB metals is concentrated in certain countries with unstable political environments such as the Congo. Therefore, social safety issues in the supply chain, such as corruption, child labor, and occupational toxins, among other issues, are worthy of future investigation.^[^ ^108^ ^]^ For instance, how can the increasing demand for raw materials affect the LIB supply chain, especially for EOL LIB practices and circular economic systems?

Below is a discussion of the future environmental influence on the supply chain in general and the CLSC in particular. The internal organizational environment and the external environment will affect each other.^[^ ^109^ ^]^ As the application of science and technology advances and organizational factors change, the external environment will also evolve.

#### Future Environment (GRQ6)

Safety issues will arise as battery recycling and recovery grow and CLSC stages mature.^[^ ^110^ ^]^ What impact will these expected increases in LIB use and recycling have on the current supply chain? What kind of safety problems will arise? How do safety issues in the EOL LIB management affect the economic benefits of the whole supply chain and circular economy, as these issues continue to arise? What type of resources, capabilities, and support will be required in the future?

## 5 Discussion

These sets of research questions have transdisciplinary implications in both practical and research fields. A great deal can be learned about CLSC management of LIB safety by trying to answer the specific and general research issues identified in the previous sections. In the short term, addressing these research questions may result in fewer safety incidents associated with LIBs. In the long term, safer CLSC and circular economy practices and activities are likely to reduce the exploitation and utilization of limited metal resources, increase the economic values of LIBs, improve the efficiency of supply chains, and result in broader social and environmental improvements. Below are some scholarly and practical implications arising from the identified safety issues, as well as for research directions that are defined in this paper.

### 5.1 Scholarly Implications

Safety issues affect the supply chain and closed‐loop supply chain activities in many ways. The proposed GRQs inspire investigation to improve supply chain safety concerns in general, but especially for end‐of‐life lithium‐ion battery activities. Investigating safety issues in the LIB supply chain requires a multidisciplinary perspective, including materials science, supply chain management, and fire protection engineering, as typical disciplines.

This study initially demonstrates the risks associated with battery material properties and structural design in LIBs. One foundational question is how solid electrolytes can improve battery safety Another large question is ways in which nascent technologies can be applied to battery design, including designs for new sensors, fuses, battery casings, or storage containers. It is evident that advances in battery design are likely to have a positive impact on the safety of the LIB supply chain, but only if safety concerns are balanced with battery performance, especially for EOL management. The SRQs and GRQs provide initial directions for materials science research strategies on LIB design, intended to reduce safety risks across the supply chain, The research questions are plentiful to keep transdisciplinary teams of technological and materials researchers busy for years.

Second, this study provides research directions for supply chain management researchers. From the perspective of academic research, a study on reducing existing risks and preventing future risks is proposed for the development of long‐term CLSC for EOL LIB practices. New technology implications within the supply chain may improve the safety and efficiency of the collection, testing, and classification processes for EOL LIBs. For example, transparent and effective information management, by using big data analysis and blockchain technology, can greatly benefit supply chain organizational and external environmental relationships for safe management of EOL LIBs. Various information and sensor technologies can help acquire significant data that can then be analyzed to propose ways to mitigate safety risks along the EOL stages of the LIB supply chain. Changes in the supply chain will also alter economic, environmental, and social benefits; the implications and tensions associated with these various organizational and managerial performance dimensions need to be balanced against safety concerns. Holistic evaluation is required across safety dimensions, general business and sustainability performance measures, and supply chain stages, especially the end‐of‐life closed‐loop supply chain stages that can greatly impact environmental and societal capital.

Finally, most safety concerns mentioned in this study are physical risks in the form of fires and explosions. Therefore, research significance extends to hazards, emergency planning, and fire protection engineering. For example, source analysis of risk across supply chain activities can improve fire protection from EOL LIB deficiencies. The multiple SRQs and GRQs can provide academic and practical significance for fire‐protection equipment, operation methods, storage and collection facility design, and supervision policy.

Each of these research streams deals with social sustainability because of the safety of workers and the safety of communities. This social sustainability research includes technology for social good and expanding social sustainability to circular economy and supply chain concerns. The linkage of social sustainability to environmental and economic sustainability concerns through safety is a significant area in need of research. EOL LIB CLSC research can extend the sustainability research in each of these directions.

### 5.2 Practical Implications

The research awareness and questions raised in this study can provide value to LIB and EOL LIB practice. Future solutions to the research questions and barriers to practical applications are possible with the use of the framework by different stakeholders in the LIB CLSC.

First, this study provides organizations and their management with initial high‐level insights for improving supply chain safety in general. Although the framework and research questions are more directly applicable to end‐of‐life LIB and in the supply chain, loop‐closing activities, the dimensions and sub‐dimensions can be applied in traditional, forward supply chain activities. The framework also sets the stage for creative ways to reduce safety problems from an internal organizational perspective. Companies and managers can analyze the financial cost and material and human resources needed to mitigate safety concerns. Tangible and intangible issues can also be evaluated in this context as well, where organizations can tie their performance systems to many of these dimensions and explicitly include the focus in their environmental, health, and safety (EHS) functions.

Second, the government plays an important stakeholder role in improving supply chain safety from an external environment perspective. The study demonstrates risks associated with LIB supply chain safety standards and ways that the government and non‐government organizations could be included to promote LIB safety policies within the supply chain, generally, and at the EOL, specifically. Previous studies have led to the identification of problems that flawed regulations cause for safety in the LIB industry. Issues include variations in standards such as LIB labeling in different countries and regions, given that the manufacture, consumption, and recycling of LIBs are international activities. The research questions presented can help promote regulations and oversight systems that are missing in practice, both locally within organizations and broadly across global supply chains and circular economies that rely on managing end‐of‐life waste streams as part of broader sustainability efforts.

Third, technology is important for practical reductions of safety problems. LIBs and general supply chains have been adopting new technologies at different stages of a product's life cycle. These technological advances are likely to be a prerequisite for supporting future safety improvements. The advantage of applying novel technical solutions should be recognized, but practical barriers to their adoption, implementation, and management should not be ignored. The rapid changes and developments of the LIB supply chain make the application of technology subject to many uncertainties and risks. Many of the emerging multi‐stakeholder information technologies can improve LIB safety throughout its lifecycle. Organizations need to anticipate and understand potential benefits and risks of applying these new technological solutions, whether they are materials, products, or process technologies. Researchers, managers, and technology providers need to work together—*trans‐disciplinarily*—to further enhance and develop technologies and overcome the multitude of barriers.

Overall, raising awareness of the links between research and practice related to safety is the first step. The research agenda provides practical, applied concerns that need to be addressed across all areas of the TOE framework.

## 6 Conclusion

The objective of this study is to provide insights and develop a research agenda for safety problems in the CLSC of LIBs, especially at the end‐of‐life stage. The study offers potential research directions for mitigating emergent safety issues associated with LIBs. The current and potential future industrial situation concerning LIB safety crises and opportunities for supply chain safety has been provided, with substantive evidence from published literature. Specifically, this research investigates LIB supply chain safety concerns with linkage to societal, environmental, and economic dimensions.

The LIB supply chain and recycling activities are core issues within a circular economy, where safety issues have been overlooked. Within this context, this research seeks both to contribute to LIB recycling safety concerns and to add to broader concerns associated with safety in a circular economy, closed‐loop supply chains, and supply chain management in general. Safety is an important social sustainability dimension that has received less overall attention than economic or environmental sustainability; although, they are all interlinked.

LIB CLSC safety concerns are classified according to four main dimensions. These dimensions are then restructured within a TOE framework, where safety factors are recategorized from the view of potential solutions and research questions for each safety issue. Specific research questions are extended to groupings of general research questions within the TOE framework, focusing on transdisciplinary opportunities for EOL LIB CLSC research and practice.

The limitations of this study are relatively evident. A research agenda and a multitude of research directions are given; the difficulty arises in prioritizing these opportunities for future research. This is not presented here, given that different research fields are involved. This study only describes the safety factors of the LIB CLSC in a general way and does not specifically point out how problems occur in different process steps. More nuanced evaluations, conducted empirically, analytically or conceptually, are needed. This study is exploratory and nascent, seeking to analyze safety issues from the perspective of supply chain management. It focuses on safety factors that cause accidents such as fires and explosions. However, as the LIB CLSC changes, there will be more operational, social, environmental, market, and other risks that deserve further study. Finally, although significant literature was used to help develop insights and evidence, this study does not provide a detailed and structured literature review of the topic, which is necessary for the guidance of a strong foundation for future studies.

Future research could be carried out from the three perspectives of technology, organization, and environment, according to the research directions proposed in the paper. Each of these individual areas can contribute to understanding and improving safety in the LIB CLSC. Future research directions could involve any or a mixture of the various research questions posed. CLSC, the circular economy, and the application of EOL management in LIBs are interrelated and necessary for LIB recycling and contribution to a more sustainable world across multiple dimensions. Safety concerns can serve as a barrier and alleviating this barrier can enhance acceptance and broader adoption of LIBs for a more sustainable and circular world.

## Conflict of Interest

The authors declare no conflict of interest.
